# The Resistance Mechanisms of Checkpoint Inhibitors in Solid Tumors

**DOI:** 10.3390/biom10050666

**Published:** 2020-04-25

**Authors:** Evangelos Koustas, Panagiotis Sarantis, Athanasios G. Papavassiliou, Michalis V. Karamouzis

**Affiliations:** 1 Molecular Oncology Unit, Department of Biological Chemistry, Medical School, National and Kapodistrian University of Athens, 11527 Athens, Greece; vang.koustas@gmail.com (E.K.); panayotissarantis@gmail.com (P.S.); papavas@med.uoa.gr (A.G.P.); 2 First Department of Internal Medicine, ‘Laiko’ General Hospital, Medical School, National and Kapodistrian University of Athens, 11527 Athens, Greece

**Keywords:** solid tumors, cancer, immunotherapy, resistance mechanism

## Abstract

The emergence of cancer immunotherapy has already shown some remarkable results, having changed the treatment strategy in clinical practice for solid tumors. Despite these promising long-term responses, patients seem to lack the ability to respond to immune checkpoint inhibitors, thus demonstrating a primary resistance to immunotherapy. Moreover, a significant number of patients who initially respond to treatment eventually acquire resistance to immunotherapy. Both resistance mechanisms are a result of a complex interaction among different molecules, pathways, and cellular processes. Several resistance mechanisms, such as tumor microenvironment modification, autophagy, genetic and epigenetic alterations, tumor mutational burden, neo-antigens, and modulation of gut microbiota have already been identified, while more continue to be uncovered. In this review, we discuss the latest milestones in the field of immunotherapy, resistance mechanisms against this type of therapy as well as putative therapeutic strategies to overcome resistance in solid tumors.

## 1. Introduction

The concept of immune therapy to fight against cancer was first described in 1890 by W. Colley, who observed cancer remissions after inoculating sarcoma patients with erysipelas cultures [1]. Significant progress has been made since then and has provided an in-depth understanding of tumor biology and interactions within the immune system. Further knowledge of the relationship between the immune system and cancer can be derived under the scope of a process named immune-editing, which comprises three distinct phases: elimination, equilibrium, and escape [2]. An intact immune system is capable of recognizing cancer cell’s antigens as “non-self", and thus unleashing sufficient immune responses in order to induce elimination. Cancer cells surviving the elimination phase may rest in a dormant situation, unable to progress under the opposing forces of immune cells. In the escape phase, cancer cells are capable of altering their main characteristics with a loss of immunogenicity and/or antigenicity. Additionally, malignant cells can gain additional immunosuppressive properties, such as expression of programmed death-ligand 1 (PD-L1) or secretion of suppressive cytokines, which result in an additional reduction of their immunogenicity [3,4].

The principal mode of action of the immune system is the recognition by cells of the innate immune system, the release of various cytokines, and complement activation and concomitant phagocytosis. Tumor development is associated with cytotoxic innate and adaptive immune cells, but as the tumor evolves from neoplastic tissue to detectable tumors, tumor cells use different mechanisms in order to create an immunosuppressive recapitulate peripheral immune tolerance [5].

This immune activation is under strict control by inhibitory checkpoints that prevent overstimulation, resulting in autoimmunity and extensive damage to healthy cells [6]. Unfortunately, tumor cells develop resistance mechanisms in order to avoid the presence of an efficient immune system. Tumor cells escape from immune response through two main mechanisms: avoiding the immune-cancer cell recognition and triggering an immunosuppressive tumor microenvironment (TME). Firstly, cancer cells rapidly decrease the expression of tumor antigens on the cell surface, thus avoiding being recognized by cytotoxic T cells. Moreover, tumor cell-derived factors initiate an immune-tolerant TME through (i) secretion of inhibitory checkpoint molecules (ii) induction of the recruitment of myeloid-derived suppressor cells (MDSCs), tumor-associated macrophages (TAMs) and regulatory T cells (Tregs) and (iii) expression of small suppressive molecules [4,7]. In order to evade immune destruction, tumor cells express inhibitory checkpoints that induce immune suppression, a mechanism that has been thoroughly investigated, which leads to the development of several molecules able to restrain cancer-induced immuno-suppression (i.e., anti-CTLA4, anti-PD1, and anti-PDL1). These immune checkpoint inhibitors now form a new landscape in cancer therapeutics, having shown remarkable response and survival rates in a variety of tumors. However, many cancer patients do not derive benefit from immune checkpoint inhibitors, an observation that has led to the assumption that resistance to immunotherapy may be present [8,9].

Resistance to immunotherapy can be categorized as primary and acquired, depending on the presence or absence of initial response and subsequent relapse after the response that has been achieved. The mechanisms involved in the development of resistance include tumor-cell intrinsic and extrinsic factors that can either interfere with the antigen processing and presentation or enable tumors to recruit immune-suppressing cells that antagonize the activity of effectors cells [6]. With the advent of the immune checkpoint blockade, cancer therapeutics shifted from a tumor-cell focused approach to a broader concept of factors contributing to tumor formation. This has led to the recognition of the tumor microenvironment not only as a significant player supporting tumor growth and metastasis but also as a contributing one to the development of therapeutic resistance. In the context of immunotherapy, TME is regarded as a critical mediator of tumor-induced immuno-suppression through a variety of mechanisms, resulting in the down-regulation of both the effector T-cell activity and the recruitment of immunosuppressive cells [10].

## 2. Immunotherapy in Solid Tumors

The significant advance of the checkpoint inhibitors (anti-CTLA-4, anti-PD-1/PD-L1) has indicated as a hopeful therapeutic option in several solid tumors. CTLA-4 antibodies, like ipilimumab and tremelimumab, interrupt CTLA-4 from interacting with B7 and boost T-cell activation. The expression of CTLA-4 on tumors has been linked with poor survival in nasopharyngeal carcinoma [11] and increased survival in non-small cell lung cancer (NSCLC) [12]. The expression of PDL1 on tumor cells is high. On ligand binding by PD-L1, the PD-1 receptor inhibits the activation and proliferation of T-cells through a phosphatase, SHP-2, which de-phosphorylates the antigen receptor expressed by these cells. High PD-1/PD-L1 levels may associate with poor prognosis in some cancers (melanoma, esophageal, renal cell carcinoma, ovarian cancer) and with better prognosis in others (e.g., angiosarcoma and gastric cancer) [13]. PD-1 antibodies (such as nivolumab and pembrolizumab) and PD-L1 (such as atezolizumab) break off the interaction of PD-1 with PD-L1. In the last decade, numerous checkpoint inhibitors (e.g., ipilimumab, nivolumab, pembrolizumab, atezolizumab) got approval in various solid tumors such as melanoma, lung, renal, and bladder [14].

More specifically, in 2011, ipilimumab was the first approved checkpoint inhibitor, in patients with metastatic melanoma (shows better overall survival-OS). Pembrolizumab and nivolumab were also among the approved PD-1 inhibitors for advanced melanoma. Synergistic therapy with ipilimumab plus nivolumab led to better PFS compared to each drug being used separately (11.5 vs. 2.9 months only with ipilimumab and 6.9 months only with nivolumab) [15].

The first immunotherapy permitted (2015) was nivolumab for the treatment of patients with metastatic NSCLC. Between patients with advanced non-squamous NSCLC that had progressed during or after platinum-based chemotherapy, OS was better by 41% (nivolumab vs. docetaxel) [16]. Moreover, first-line treatment with nivolumab along with ipilimumab resulted in a better OS than only chemotherapy, in patients with NSCLC, independent of the PD-L1 expression [17].

Furthermore, the combination of nivolumab plus ipilimumab versus sunitinib shows significantly better OS rates among intermediate and poor-risk patients with previously untreated advanced renal-cell carcinoma [18].

Pembrolizumab was also used for second-line treatment of patients with NSCLC that express PD-L1. Additionally, it is authorized for the handling of patients with advanced NSCLC, high PDL1 expression, and metastatic melanoma patients and patients with recurrent/metastatic of the head and neck squamous cell carcinoma [14].

Atezolizumab (PD-L1 inhibitor), is already used for the treatment of patients with locally advanced or metastatic urothelial carcinoma due to its favorable safety profile compared to chemotherapy [19]. Moreover, the administration of atezolizumab versus bevacizumab plus chemotherapy significantly improved PFS and OS between patients with metastatic non-squamous NSCLC, independently of PD-L1 expression and EGFR/ALK genetic background [20].

## 3. Resistance Mechanism

PD-1/PD-L1 blockade appears to be promising immunotherapy with significant clinical benefits and durable responses in multiple tumor types. A percentage of cancer patients never demonstrate a clinical response or stabilized disease after treatment with checkpoint inhibitors. The estimated percentage of US cancer patients who are eligible for ICI (such as anti-PD-1/PD-L1, anti-CTLA4) increased from 1.54% in 2011 to 43.63% in 2018. Furthermore, the percentage of cancer patients estimated to respond to ICI was 0.14% in 2011 and increased to 12.46% in 2018 [21]. Different data from clinical studies suggest that the presence of pre-existing CD8+ T cells within the TME and the expression of PD-L1 and PD-1 on the tumor and T cells, respectively, predicted the response of patients to anti-PD-1 [22]. Therefore, several studies have highlighted the resistance mechanism against immunotherapy as a phenomenon in which CD8+ T-cells are either unable to recognize and localize to the tumor or are rendered ineffective despite their seemingly adequate localization [23]. Resistance mechanism against immunotherapy involves various mechanisms by which tumors can escape from the immune response.

### 3.1. The Immunosuppressive Mechanism in TME

The tumor microenvironment is a complicated structure that includes M2 macrophages, fibroblast, Tregs, myeloid-derived suppressor cells (MDSC), and other stromal cells [24].

Tregs are essential lymphocytes maintaining self-tolerance. This kind of T-cells is characterized by high expression of the FoxP3 transcription factor [25] and they can suppress effector T cells or (Teff) through secretion of inhibitory cytokines such as TGF-beta and/or IL-10 [26]. It is common for several kinds of tumors that are infiltrated by Tregs to depict such a pattern, and the lack of TME can restore this anti-tumor immunity [27].

A subtype of B-cells, which is known as B regulatory cells or B-regs, has been associated with autoimmunity, inflammation, and tumorigenesis [28]. B-regs are known as negative regulators of the immune response through suppression of cytotoxic CD8+/CD4+ Teffs, the secretion of anti-inflammatory cytokines (such as interleukin (IL)-10) [29,30] and the release of co-inhibitory molecules such as programmed cell death-ligand 1 or PD-L1 [31].

MDSCs are characterized by the expression of several molecules, such as CD11b and Gr-1 markers [32]. Different studies highlight the effect of MDSCs as a critical regulator mechanism of immune responses in cancer. Moreover, MDSCs regulate and promote cancer cell invasion and metastasis [32]. In addition, the presence of MDSCs in TME is correlated with resistance against immunotherapy and lower survival in different types of tumors such as colorectal and breast cancer [24].

Another essential type of white cells in TME is tumor-associated macrophages (TAMs), including M1 and M2 macrophages [33,34]. Several studies correlate TAMs with responses to immune therapies through suppression of T cell responses with PD-L1 [35]. Type M1 macrophages are associated with anti-tumor immunity and M2 with pro-tumorigenic properties [36,37].

TME is characterized by tumor-associated mast cells or TAMCs. This kind of cells is being recruited by chemotactic molecules that are secreted by cancer, immune and stromal cells such as prostaglandin E₂ (PGE2), vascular endothelial growth factor (VEGF), CXC chemokine ligand (CXCL)12, fibroblast growth factor (FGF)-2, and platelet-derived growth factor (PDGF) [38]. Activated TAMCs release IL-33 (an essential cytokine for the activation of mast cell) and the C-C motif chemokine ligand (CCL)-5 (chemotactic factor) to further support the activation of mast cells into TME [38]. Moreover, TAMCs can also release a vast array of proteolytic enzymes and growth factors in order to promote immunosuppression, angiogenesis, tumorigenesis, and cancer cell invasion and metastasis [38,39].

### 3.2. Autophagy as a Modulator Mechanism for Antigen Presentation

#### 3.2.1. The Association of Autophagy and the Immune System

Autophagy is a well-described catabolic mechanism where molecules and damaged cellular organelles are degraded [40]. The primary step of autophagy is the formation of the autophagosome, a spherical structure with double-layer membranes, which consists of cytoplasmic contents and fuses with lysosomes [41]. The role of autophagy in cancer remains controversial [42]. This mechanism is associated with anticancer therapy by shaping hypoxic, metabolic, inflammatory, and immunosuppressive tumor microenvironments (TME) [43,44]. The autophagosome endosomal trafficking is closely connected with angiogenesis, immunosuppression and chronic inflammation, stromal formation, metastatic and the metabolic potential of cancer cells of TME [43,45,46].

Several studies support the hypothesis that autophagy can alter the pool of antigenic peptides that are presented in the major histocompatibility complexes (MHC) I and II [47].

The fusion of New York esophageal squamous cell carcinoma-1 (NY-ESO-1), a specific antigen frequently over-expressed from melanoma to LC3, leads to the targeted autophagosome increasing response of NY-ESO-1-specific anti-melanoma helper T-cell [48]. Moreover, in autophagy based vaccination, autophagosomes from cancer cells are treated with proteasome inhibitors. These autophagosomes comprise tumor-associated antigens and, on the surface, express C-type lectin domain family 9 member A (CLEC9A) ligands that facilitate endocytosis by antigen-presenting cells (APCs) [49].

Dendritic cells (DCs) pulsed with such autophagosomes were more efficient in the response of OVA-specific T-cell compared to soluble protein [50]. It appears that this kind of autophagosome vaccination decreases the B16F10 melanoma cell viability, eliminated 3LL Lewis lung tumors, and preserved mice models from sarcoma [51]. Additionally, cancer cells may release autophagosomes, modulate autophagy machinery and positively alter the anticancer efficacy of T-cell in immune responses [52,53]. In a recent research article, the authors support the hypothesis that checkpoint inhibitors such as nivolumab, pembrolizumab, and ipilimumab activate the cytoprotective mechanism of autophagy in colorectal cancer cell lines [54].

#### 3.2.2. The Correlation of Autophagy and Antigen Presenting Cells

It is well known that anti-tumor T-cells are activated via the identification of tumor peptides with immunogenic activity. These peptides are presented on the surface of antigen-presenting cells, such as DCs. Several studies highlight the essential role of the autophagy machinery in the regulation of the major histocompatibility complex (MHC) I and the antigen presentation on the surface of APCs such as macrophages and dendritic cells. In embryo mice, the expression of MHC class I on the cell surface of APCs was increased after treatment with autophagy inhibitors or knockdown of essential genes for autophagy regulation [55,56]. In a recent study, observed that slower trafficking of MHC-I consequently augmented CD8+ T cell activation [56]. Hence, due to a lack of autophagy initiation, observed higher expression and lower degeneration speed of MHC-I [51]. In vivo models, the absence of vacuolar protein sorting-associated protein 34 (VPS34) increased MHC-I and II expression on the DC surface [57]. In contrast, the expression of MHC-II on the cell surface of the macrophages was down-regulated after the inhibition of autophagy initiation with 3-methyladenine (3-MA) [51].

Several studies have highlighted the association of autophagy with the cross-presentation (the loading of MHC-I into APCs with immunogenic peptides) of antigens in DCs [43]. Increasing levels of autophagy were associated with the cross-presentation capability of bone marrow-derived DCs [56,58]. Moreover, the presentation of antigen in MHC-II was altered after autophagy inhibition with reduction of DC treatment intervened by an immune-dominant mycobacterial peptide with the reduced presentation of the vaccinia virus Ankara antigens and herpes simplex virus (HSV) antigens [59,60]. Thus, the initiation of T-cell response through antigen-specific peptides was decreased. So autophagy inhibition with specific agents altered the pool of immunogenic peptides, which is loaded in MCH and decreased the presentation of immune-dominant epitopes [61]. While autophagy inhibition increases the expression of MHC-I on the cell surface, it can alter the pool of peptides, which are loaded on MHC of DCs surface [43,62,63].

### 3.3. Genetic/Epigenetic Alteration in Cancer

Several studies support the hypothesis that epigenetic alteration with DNA methyltransferase (or DNA MTase) and histone deacetylase (or HDAC) inhibitors as a putative target that increases the efficacy of ICI. These types of inhibitors increase the production of cytokines. They also increase antigen presentation by inhibiting Tregs [64]. In a recent study using a mouse model with ovarian cancer, inhibitors against DNA MTase augmented the production of immune-stimulatory chemokines such as CXCL9 or 10, and re-sensitized cancer cells to anti-PD-L1 MoAbs [65]. Moreover, in CRC and mammary carcinoma models, inhibition of histone deacetylase and DNA methyltransferase enhance response when combined with anti–CTLA-4 and anti–PD-1 checkpoint inhibitors by downregulating MDSCs [66].

### 3.4. Tumor Mutational Burden

In a number of patients, the residual capacity of the immune system can be restored through exogenous tumor-infiltrating lymphocytes (TILs) or T-cells directed to cancer-specific immunogenic peptides. Although cancer cells are evolving in order to trigger antimmunosuppressive environment. This leads to genetic and/or epigenetic alterations that interrupt the formation of neo-antigen [67]. Consequently, this leads to antigens’ inability to be recognized by cytotoxic lymphoid cells such as T-cells and decrease the sensitivity of tumor cells to immunotherapy, as it happened for cancer patients with large diffuse B-cell lymphoma; as a result CD19 loss of expression [68]. Furthermore, acquired resistance against immune checkpoint inhibitors (ICI) and Adoptive cell transfer (ACT) is developed through genetic alterations but also cancer cells and inhibition of antigen-immunogenic peptides complexes, as well as the activation of cytotoxic T-cell [69,70,71]. Several studies highlighted the positive correlation between the number of neo-antigens and high mutational burden with the response to ICIs [72,73,74]. The lack of cancer cell sensitivity to immunotherapy may also be the result of the abnormal growth of cancer cell clones within the tumor that do not produce neo-antigens [67]. Thus, the construction of genetically-modified T-cells with increased longevity remains the main target to avoid the resistance against immunotherapy.

### 3.5. Molecular Mechanisms as Immunosuppressive Mechanisms

Several molecular mechanisms and oncogenes have been associated with resistance to immunotherapy in solid tumors.

The microphthalmia-associated transcription factor (MITF) is a well-known oncogene of melanoma, which is negatively associated with the invasion properties of cancer cells [75]. The MITD-depended resistance has already been described. Inhibition of eIF2B activates ATF4 and consequently AXL and suppresses MITF. This leads to resistance in adoptive T-cell and anti-PD-1 Monoclonal Antibodies (MoAbs) [76]. Furthermore, in melanoma, histone methyltransferase Ezh2 regulates the T-cell resistance against immunotherapy [77]. Ezh2-PRC2 protein complex regulates T-cell infiltration in melanoma. In a mice model, anti-CTLA4 MoAbs or IL-2 led in T-cells and TNF-a accumulation and Ezh2 expression, which caused immunogenicity loss of tumor, lower antigen expression and resistance to immunotherapy. Activation of Ezh2 in the same model re-established the immunogenicity of the tumor and inhibited melanoma growth [78]. Another crucial mechanism that affect ICI-based therapy are MicroRNA (miRNAs). miRNAs control tumorigenesis through the regulation of different genes and act as a resistance mechanism against immunotherapy [E30]. In melanoma, several miRNAs reveal acquired resistance to MAPK inhibitors as well as innate resistance to anti-PD-1 checkpoint inhibitors, which are both related to alterations of inflammatory and angiogenic pathways [78]. Other studies in melanoma and lung cancer support that miR-8 family target PD-L1 leads to an increase in the effectiveness of CD8^+^T-cell and tumor cell immunosurveillance. The increasing levels of the PD-L1/PD-1 molecules may be the result of the decreasing production of these miRNAs [79,80].

The role of signal transduction pathways in cancer is well-described in many studies. In a melanoma animal model, the lack of phosphatase and tensin homolog (PTEN) activation decreased the infiltration of T-cells in tumors and increased the expression of immunosuppressive cytokines. Furthermore, VEGF was increased and led to immune suppression as it was highlighted through clinical samples and B16 murine tumors [81,82]. Moreover, loss of PTEN leads to autophagy inhibition through activation of the phosphoinositide 3-kinases (PI3K) signaling pathway, which had little effect on T-cells and immunotherapy [83,84]. In other human studies, the association between PTEN loss and resistance against PD-L1 MoAbs pembrolizumab was further confirmed [85,86].In addition, mutations in the JAK1/2 signaling pathway lead to resistance against anti-PD-1 MoAbs [87,88]. In colorectal cancer (CRC) and melanoma patients with deficient mismatch repair (dMMR) system and resistance against anti-PD-1, several mutations in JAK1/2 were detected [87]. Another study also supports the hypothesis of the correlation between resistance to anti-PD-1 MoAbs and mutation on JAK1/2 [89]. Dysfunctional JAK1/2 leads to downregulation or lack of expression of PD-L1 through inactivation of the IFN-γ receptor pathway and resistance against anti-PD-1/PD-L1 MoAbs [78]. The well-described Wnt signaling pathway is associated with several diseases, including cancer [90]. In a recent study, the authors highlighted the correlation between the WNT/β-catenin axis and the loss of T-cell gene expression in metastatic melanoma samples [91]. Moreover, in metastatic melanoma, through immune exclusion, caused by deficient recruitment of dendritic cells (DC CD1031), the activation of the Wnt signaling pathway leads to the immunotherapy resistance of cancer cells against anti-PD-L1 and anti-CTLA-4 ΜοAbs [91,92].

Last, beta-2-microglobulin (B2M) is correlated with the heavy chain of MCH- I [93]. Mutations, deletion, or loss of heterozygosity harbored in this gene were detected in almost 30% of melanoma patients treated with ICI. Moreover, a mutation in B2M gene may alter the antigen presentation on MHC I and enrich three-fold in non-responders (30%) compared to responders (10%) along with more reduced OS in independent cohorts of patients with melanoma treated with anti-CTLA4 and anti-PD1 MoAbs respectively [94].

### 3.6. The Relation between Gut Microbiota and Immune Response

In the last decade, the association between gut microbiome and response to immune ICI is gaining interest. It is well-established that diet may alter the microbial composition. A study supported the hypothesis that fiber can trigger an immunostimulatory microbial landscape, which may lead to an increased response to ICI [95]. Moreover, probiotics may help to reshape the microbiome when they combine with ICI positively. In a melanoma mouse model, Bifidobacterium showed a synergistic effect with a PD-L1 checkpoint inhibitor [96].

**Figure 1:**
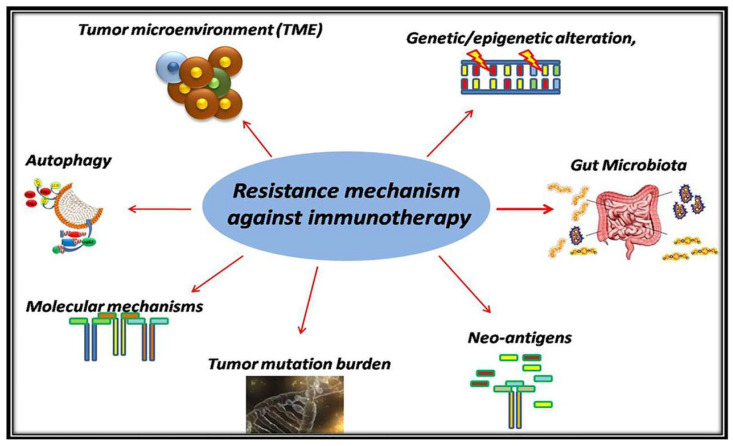
The predominant mechanisms of immunotherapy resistance in solid tumors. Several potential tumor-related mechanisms have already been identified as resistance mechanism against immunotherapy. Tumor microenvironment through the complexity of its structure, autophagy–depended antigen presentation on MHC I/II of antigen-presenting cells (APCs), tumor mutation burden and genetic/epigenetic alteration, molecular mechanism such as mutation several genes are the main mechanism of resistance in solid tumors.

Moreover, several studies highlighted the correlation between higher bacterial diversity (such as *Faecalibacterium* and *Ruminococcaceae*) enrichment of specific species and improved response to ICI [9,97]. The gut microbiome appears to modulate responses to anti–PD-1 checkpoint inhibitors in melanoma patients [98]. A recent study revealed that germ-free mice with fecal transplants from responders to ICI developed improved outcomes with anti–PD-L1 checkpoint inhibitors [99]. It is well known that antibiotics can alter the response to ICI through the modification of individual species [9,100,101,102]. The correlation between ICI response and microbiota is likely, via cross-reactivity between tumor neo-antigens and gut microbial, augmenting DC response, antigen presentation and the production of inflammatory cytokine [103,104]. In light of these results, several clinical trials have focused on investigating the influence of microbiome to immunotherapy response [105]. The predominant mechanisms are summarized in Figure 1.

## 4. Ways to Overcome the Resistance Mechanism Against Checkpoint Inhibitors

In recent years, the field of immune-oncology has established an increased understanding of molecular behavior of cancer, leading to the development of several therapeutics strategies, based on re-activation of immune system, against solid tumors. Despite the demonstrated successes of checkpoint inhibitors (ant-PD-1, anti-PD-L1, ant-CTLA4 etc.), most patients with solid tumors do not respond.

It is a common belief that PD-L1 expression in tumor cells immunohistochemistry (IHC) with the Tumor Proportional Score (TPS) is the only checkpoint inhibitor that is used as a predictive biomarker approved for NSCLC patients in first- and second-line treatment [106,107]. Unfortunately, checkpoint inhibitors against PD-1/PD-L1 have not been shown to play an essential role in predicting the immune response in other solid tumors or different settings. Moreover, the lack of PD-L1 expression in several cancers (as a biomarker), at a single time point may not fully represent the complexity of cancer cell communication network within TME [108,109].

The last years, research efforts revealed the complex and highly heterogeneous structure of TME. As it was mentioned before in the current review, TME is a main resistance mechanism against ICI. The following can be used to reduce the resistance of TME: (a) Upregulation of chemokines (CXCL) 9 and 10. Doxorubicin may induce the activity of CXCL10. The goal of a phase I/II study is to evaluate the effect of doxorubicin hydrochloride when given together with pembrolizumab in patients with sarcoma (NCT02888665); (b) activation of the endosomal toll-like receptors (TLRs) 3, 7, 8 and 9 [110]; (c) epigenetic silencing of Th1 cell-type chemokines; (d) blockade of the CXCL12/CXCR4 axis; (e) inhibition of MDSC using PI3K inhibitors;and (f) use of antiangiogenic drugs [111]. Several ongoing clinical trials try to investigate the role of antiangiogenic agents in order to enhance the effect of ICI. For example in a phase I/II study they combined lenvatinib (VEGFR inhibitor) with pembrolizumab in patients with advanced solid tumors (NCT02501096) (g) use of low molecular weight heparins (LMWHs) [112] (h) combined radiation therapy and PD-1/PD-L1 blockade, leading to an increased CD8+/Treg ratio and decreases immunosuppressive MDSCs. The investigators in a randomized Phase II clinical trial hypothesize that in a significant subset of patients with recurrent NSCLC immunotherapy (pembrolizumab) after stereotactic body radiation therapy (SBRT) (NCT02492568) will be superior to treatment with immunotherapy alone [113]. In a recent study, MHC I/II molecules appear to downregulated in resistance mutant Kras and p53-deficient lung cancer cells. However, local radiotherapy leads to increasing levels of IFN-β and MHC I molecules on the cell surface of resistant cells. Thus, it is proved that adjuvant radiotherapy may help to overcome anti-PD-1 resistance, and then enhances the efficacy of anti-PD-1 checkpoint inhibitors [114]

An increasing amount of research data supports the hypothesis that targeting the structure of blood vessels can reduce the function of suppressive cells and promote the anti-tumor activity of immune effector cells within TME [115]. Currently, a plethora of clinical studies are underway in order to identify the impact of simultaneous inhibition of angiogenesis and checkpoint inhibitors. Moreover, many research teams are focusing on reprogramming TME in order to become more immune-stimulatory through a therapeutic scheme that combines anti-angiogenic agents and immune checkpoint inhibitors such as pembrolizumab and nivolomumab. In this contest, this combinatorial scheme appears to inhibit the negative immune signals and augment the ratio of anti-/pro-tumor immune cells. Furthermore, immunotherapy appears to restore immune-supportive TME and promote tumor-vascular normalization. These facts lead to an increase in the infiltration and activation of lymphocyte within the tumor [115].

In addition, several studies highlight the fact that anti-PD-L1 based immunotherapy appears to be more efficient when combined with chemotherapeutic agents [43]. On the other hand, different cancer types, such as breast or colorectal cancer, and melanoma, are identified through the higher expression of PD-L1 in both cancer and infiltrating immune cells [116]. Further, ongoing clinical studies are trying to evaluate the combinatorial scheme of anti-PD-1/-L1, MoAbs with other therapeutic agents such as copanlisib (PI3Kinase inhibitor) in elapsed/refractory solid tumors with expansions in mismatch-repair proficient (MSS) colorectal cancer patients (NCT03711058). In addition, other clinical trials combine platinum-based agents such as carboplatin with nivolumab in order to evaluate the pathological complete response defined as the absence of residual tumor in lung and lymph nodes comparing patients treated with chemo-immunotherapy versus chemotherapy alone. FOLFOX scheme also has been combined with ICI in several clinical trials (NCT03202758, NCT02375672, and NCT02997228). In a randomized phase 2 study will evaluate 2 novel immunotherapy combinations in which pembrolizumab is integrated with ramucirumab and paclitaxel in patients with advanced gastric and GEJ adenocarcinoma (NCT04069273) The main goal of this study is to examined the re-activation of the immune response against several types of cancer with therapeutic benefits for patients.

In addition, pembrolizumab as monotherapy could better suit a patient with a low tumor burden and a better performance status, than the combination of pembrolizumab and chemotherapy that would be more beneficial for a cancer patient with a higher tumor burden and a more inferior performance status, for whom a rapid and more probable response to treatment is crucial [117]. Furthermore, this very same problem could also arise with another anti-PD-1 drug, atezolizumab, apart from the positive results coming from the IMpower150 clinical trial. Moreover, atezolizumab also showed interesting results in a recent study from the IMpower110. In this study, 555 high PD-L1 expressing (TC3/IC3) naïve nonsquamous or squamous advanced NSCLC-affected patients without positive genetic biomarkers were respectively randomized (1:1) to receive atezolizumab monotherapy vs. cis/carboplatin + pemetrexed or atezolizumab monotherapy vs. cis/carboplatin + gemcitabine and results decisively favored atezolizumab over SoC chemotherapy: mOS: 20.2 vs. 13.1 months (HR for death: 0.595) [117].

The crucial role of autophagy as a regulator mechanism for energy and metabolic balance in tumor cells is well described previously. The last decade research efforts have led to the development of agents that modulate autophagy. Chloroquine (CQ) and its derivative, hydroxychloroquine (HCQ), is one of the most studied inhibitors that target the fusion of the autophagosome with a lysosome [43]. Several studies indicate that autophagy inhibition in cancer cells may be a putative an approach to improve the effect of ICI. High-dose IL-2 (HDIL-2) alone has been found to be beneficial for immunotherapy in an advanced murine metastatic liver tumor model especially after co-treatment with CQ. It is known that IL-2 reduces tumorgenesis through initiation of immune cell proliferation and infiltration in the liver and spleen [118]. In another study in renal cell carcinoma, autophagy inhibition by CQ also increased the effect of HDIL-2 on stimulation of T-cells, NK cells and DCs [119]. In addition, combination of 3-MA and IL-24 induced apoptosis in oral squamous cell carcinomas (OSCC) [120]. Furthermore, as it was mentioned before in the current review, ICI such as nivolumab, pembrolizumab or ipilimumab can trigger the cytoprotective autophagy in CRC cell lines. The combination of Hydroxychloroquine or HCQ (autophagy inhibitor) and checkpoint inhibitors trigger apoptotic cell death in MSHI-H CRC cell lines [54]. The clinical response of CQ and it derivative HCQ appears to vary widely. Both of them are not specific inhibitors of autophagy and affect also other cellular functions [43]. Thus, a plethora other agents that modulate autophagy (inhibitors or promoters) have already been developed [43]. The impact of autophagy on tumorgenesis and its active participation in antigen presentation from MHC-I and/or MHC-II make autophagy an attractive target for solid tumor ICI-depended therapy.

As it was mentioned, mutation in different genes and the signaling pathways that control have been targeted from many research teams in order to overcome the resistance against ICI. The phase I/II trial studies (NCT04317105) tries to investigate the side effects and best dose of copanlisib (dual inhibitor of PI3K α and δ isoforms) when given together with nivolumab and ipilimumab in treating patients with solid cancers that have spread to other places in the body (advanced) with mutation in PIK3CA and PTEN genes. A novel small inhibitor CGX1321 (inhibitor of WNT pathways) has already entered in human clinical trials as an anti-cancer agent. Keynote 596 uses single agent dose expansion phase in gastrointestinal (GI) tumors and roll-over cohort of CGX1321 and pembrolizumab in subjects who have progressed on single agent CGX1321 and Phase 1b consisting of CGX1321 in combination with pembrolizumab in colorectal tumors. Both phases of this study try to evaluate the safety, pharmacokinetics, and clinical activity of this combinatorial treatment (NCT02675946)

In addition, several inhibitor of the histone lysine methyltransferase EZH2 such as CPI-1205 are already developed. In a Phase I/II clinical study (NCT03525795), combine CPI-1205 with ipilimumab in patients with histologically or cytologically confirmed advanced solid tumors. To avoid this resistance caused by gut microbiota, antibiotics, prebiotics (dietary or chemical entities), and synbiotics can be used.

Moreover, the food industry have been applied recently bacteriophages, to eliminate unfavorable bacteria [121]. Furthermore, gut microbiota have begun to attract many research team as putative target for solid tumors. In clinical trial with clinicaltrials.gov: NCT03829111 the investigators try to combine checkpoint inhibitors such as nivolumab and Ipilimumab with probiotics. This phase I trial study tries to investigate the effect of CBM588 probiotic in patients with kidney cancer (stage IV) that are treated with nivolumab and ipilimumab.

In Table 1 are presented several ongoing clinical trials that try to combined checkpoint inhibitors with other agents in order to overcome the resistance against ICI.

## 5. Conclusions

In recent years, the vigorous attempt in the field of oncology and immunology has produced an increased amount of knowledge in tumor biology, leading to the development of several therapeutic strategies linked to the immune system. Despite the established successes of immunotherapy in solid tumors, a remarkable amount of patients with different types of cancers do not respond. Several mechanisms such as tumor microenvironment, immune cell infiltration within the tumor, poor antigen presentation, autophagy, molecular mechanisms, and silence of the immune response through cytokine release have already been identified as immunosuppressive factors in solid tumors. However, additional mechanisms continue to be discovered and further reveal the complexity of interactions between the tumor and the immune system. Myriad of research studies and clinical trials try to overcome the resistance mechanism against immunotherapy through the combination of anti-PD-1/PD-L1 or other checkpoint inhibitors with anti-angiogenic factors with traditional chemotherapy. In the future, research efforts should focus on the understanding of tumor microenvironment structure in order to re-active and sensitize tumor cells on immunotherapy. Putative therapeutic strategies will be developed in order to overcome the anti-tumor immune resistance against immunotherapy for different types of cancer.

## Figures and Tables

**Table 1:** Clinical studies with combination of immunotherapy with chemotherapy in solid tumors.

Number of Study.	Type of Cancer	Phase	Agent/Compound
NCT04069273	Adenocarcinomas of the esophagogastric junction	II	Ramucirumab + pembrolizumab + paclitaxel
NCT02501096	Advanced solid tumors	I/II	Lenvatinib + pembrolizumab
NCT02646748	Advanced solid tumors	Ι	Pembrolizumab+INCB combinations
NCT04317105	Advanced malignant solid neoplasm	I/II	Copanlisib, ipilimumab, nivolumab
NCT03525795	Advanced solid tumors	I/II	CPI-1205, ipilimumab
NCT02617589	Brain cancer	III	Nivolumab, temozolomide
NCT02684006	Clear cell	III	Avelumab + axitinib vs. sunitinib
NCT02853331	Clear cell	III	Pembrolizumab + axitinib vs. sunitinib
NCT01472081	Clear cell/non-clear cell	I	Nivolumab + sunitib/pazopanib
NCT02420821	Clear cell, sarcomatoid	III	Atezolizumab+bevacizumab vs. sunitib
NCT03202758	Colorectal cancer	I/II	Durvalumab, tremelimumab and FOLFOX
NCT02981524	Colorectal cancer	II	Cyclophosphamide followed by Pembrolizumab
NCT03657641	Colorectal cancer	I/II	Pembrolizumab + vicriviroc
NCT02375672	Colorectal cancer	II	Pembrolizumab + FOLFOX
NCT03711058	Colorectal cancer	I/II	Nivolumab + copanlisi, nivolumab
NCT02327078	Colorectal cancer	VII	Nivolumab + epacadostat
NCT03832621	Colorectal cancer	II	Nivolumab, ipilimumab, temozolomide
NCT02675946	Gastrointestinal cancers	I	CGX1321+pembrolizumab
NCT02496208	Genitourinary tumors	I	Cabozantinib + nivolumab/ipilimumab
NCT02997228	mCRC	III	Atezolizumab + bevacizumab + mFOLFOX6
NCT01950390	Melanoma	II	Ipilimumab + bevacizumab
NCT02802098	Metastatic breast cancer	I	Durvalumab+ bevacizumab, taxane+ bevacizumab
NCT00790010	Metastatic melanoma	I	Ipilimumab, bevacizumab
NCT02959554	Metastatic renal cell carcinoma	II	Nivolumab after sunitinib/pazopanib
NCT03149822	Metastatic renal cell carcinoma	I/II	Pembrolizumab + cabozantinib
NCT02681549	mNSCLC	II	Pembrolizumab + bevacizumab
NCT03976375	mNSCLC	III	Pembrolizumab, lenvatinib, docetaxel
NCT03838159	NSCLC	II	Paclitaxel, carboplatin, nivolumab
NCT03425006	NSCLC	II	Itacitinib, Pembrolizumab
NCT02492568	NSCLC	II	SBRT, pembrolizumab
NCT02443324	NSCLC, Biliary tract carcinoma, Urothelial carcinoma	I	Ramucirumab + pembrolizumab
NCT03153410	Pancreatic cancer	I	Pembrolizumab, GVAX, cyclophosphamide, IMC-CS4
NCT02648282	Pancreatic cancer	II	Pembrolizumab, GVAX, cyclophosphamide
NCT03563248	Pancreatic cancer	II	Nivolumab, losartan, FOLFIRINOX
NCT03829111	Renal Cell carcinoma	I	Nivolumab, Ipilimumab, Clostridium butyricum CBM 588 probiotic strain
NCT02888665	Sarcoma	I/II	Doxorubicin+ pembrolizumab
NCT03898180	Urothelial carcinoma	III	Lenvatinib + pembrolizumab

NCT: national clinical trial; mCRC: metatstatic colorectal cancer; mNSCLC: metastatic nonsquamous non–small cell lung cancer; FOLFOX: folinic acid, fluorouracil, and oxaliplatin; mFOLFOX6: leucovorin calcium (folinic acid), fluorouracil, and oxaliplatin; GVAX: cancer vaccine which includes granulocyte-macrophage colony-stimulating factor (GM-CSF); IMC-CS4: a monoclonal antibody targeted to the colony-stimulating factor receptor (CSF-1 receptor or CSF-1R); FOLFIRINOX: 5-fluorouracil, leucovorin, irinotecan, and oxaliplatin; SBRT: Stereotactic body radiation therapy.
